# High-Accuracy Lower-Limb Intent Recognition: A KPCA-ISSA-SVM Approach with sEMG-IMU Sensor Fusion

**DOI:** 10.3390/biomimetics10090609

**Published:** 2025-09-10

**Authors:** Kaiyang Yin, Pengchao Hao, Huanli Zhao, Pengyu Lou, Yi Chen

**Affiliations:** 1 School of Electrical and Mechanical Engineering, Pingdingshan University, Pingdingshan 467000, China; 26113@pdsu.edu.cn (K.Y.);; 2 School of Intelligent Manufacturing Ecosystem, Xi’an Jiaotong-Liverpool University, Suzhou 215400, China

**Keywords:** KPCA-ISSA-SVM, sEMG-IMU fusion, locomotion intention recognition, human–machine rehabilitation devices, machine learning, nonlinear dimensionality reduction

## Abstract

Accurately decoding human locomotion intention from physiological signals remains a significant hurdle for the seamless control of advanced rehabilitation devices like exoskeletons and intelligent prosthetics. Conventional recognition methods often falter, exhibiting limited accuracy and struggling to capture the complex, nonlinear dynamics inherent in biological data streams. Addressing these critical limitations, this study introduces a novel framework for lower-limb motion intent recognition, integrating Kernel Principal Component Analysis (KPCA) with a Support Vector Machine (SVM) optimized via an Improved Sparrow Search Algorithm (ISSA). Our approach commences by constructing a comprehensive high-dimensional feature space from synchronized surface electromyography (sEMG) and inertial measurement unit (IMU) data—a potent combination reflecting both muscle activation and limb kinematics. Critically, KPCA is employed for nonlinear dimensionality reduction; leveraging the power of kernel functions, it transcends the linear constraints of traditional PCA to extract low-dimensional principal components that retain significantly more discriminative information. Furthermore, the Sparrow Search Algorithm (SSA) undergoes three strategic enhancements: chaotic opposition-based learning for superior population diversity, adaptive dynamic weighting to adeptly balance exploration and exploitation, and hybrid mutation strategies to effectively mitigate premature convergence. This enhanced ISSA meticulously optimizes the SVM hyperparameters, ensuring robust classification performance. Experimental validation, conducted on a challenging 13-class lower-limb motion dataset, compellingly demonstrates the superiority of the proposed KPCA-ISSA-SVM architecture. It achieves a remarkable recognition accuracy of 95.35% offline and 93.3% online, substantially outperforming conventional PCA-SVM (91.85%) and standalone SVM (89.76%) benchmarks. This work provides a robust and significantly more accurate solution for intention perception in human–machine systems, paving the way for more intuitive and effective rehabilitation technologies by adeptly handling the nonlinear coupling characteristics of sEMG-IMU data and complex motion patterns.

## 1. Introduction

Human motion intent recognition, as a cutting-edge research domain in biomechatronic systems and intelligent human–machine interaction, leverages biomimetic principles to decode neuromuscular signals (e.g., surface electromyography, sEMG) and kinematic features (e.g., inertial measurement unit, IMU data) through mechanisms inspired by biological proprioception and sensorimotor integration. This capability mimics the hierarchical control of natural neuro-musculoskeletal architectures, enabling assistive devices such as lower-limb rehabilitation robotics and smart prostheses to replicate human-like anticipatory adaptation during locomotion [1]. By emulating the biological redundancy and fault tolerance inherent in vertebrate motor control systems, accurate intent recognition ensures harmonious human–device interaction that parallels the safety and adaptability of natural symbiotic relationships [2]. Consequently, the development of robust and precise locomotion intent recognition algorithms remains a pivotal research focus to advance this field [3,4].

Human locomotion intent recognition methods are generally categorized into three primary types based on sensor modalities: bioelectrical signal analysis [5,6,7], kinematic modeling [8,9], and multimodal information fusion [10]. Bioelectrical approaches, which predominantly utilize surface electromyography (sEMG), employ either musculoskeletal biomechanical models or deep learning (DL) algorithms to decode neuromuscular activity. Musculoskeletal frameworks establish a relationship between sEMG signals and joint torque as well as kinematic parameters (e.g., angular velocity and acceleration) through Newton-Euler mechanics [11]. In contrast, DL models, such as recurrent neural networks (RNN) [12], convolutional neural networks (CNN) [13], and their enhanced variants [14,15], directly translate preprocessed sEMG features into locomotion patterns [16]. Despite their physiological relevance, these methods encounter challenges, including sEMG signal attenuation, low signal-to-noise ratios, and sensitivity to electrode displacement. Kinematic modeling, which combines multibody inverse dynamics with reinforcement-learning-based predictive control [17], enables robust gait phase recognition but heavily relies on accurate inertial parameters [18], thereby constraining its practical applicability.

To address these challenges, multimodal fusion strategies synergistically combine sEMG with inertial measurement units (IMUs), force-sensitive resistors, and air-pressure mechanomyography [19,20]. Current innovations span three directions: (1) Deep learning architectures, such as hybrid LSTM-CNN models (97.21% gait recognition accuracy [21]) and CNN-based frameworks enabling seamless locomotion mode transitions [22]; (2) Feature-engineering techniques, exemplified by PCA-driven BP neural network model that maintain 93.6% classification accuracy [23]; and (3) Meta-heuristic algorithms, including swarm intelligence-enhanced algorithms (e.g., GA-PSO) are used to determine the best parameters for a recognition method [24,25]. Despite these innovations, significant challenges persist when processing complex, nonlinear human locomotion data. These include how to effectively extract and select critical information from high-dimensional feature spaces and how to optimize parameters of machine learning models to achieve higher recognition accuracy and generalization capabilities.

To overcome these limitations, this study proposes a novel framework for lower-limb locomotion intent recognition by integrating kernel principal component analysis (KPCA) and a support vector machine (SVM) optimized through an improved sparrow search algorithm (ISSA). The framework exploits the complementary characteristics of sEMG and IMU data by constructing a comprehensive high-dimensional feature space and applying nonlinear dimensionality reduction to extract discriminative low-dimensional principal components. Concurrently, the ISSA facilitates precise optimization of SVM hyperparameters, thereby substantially improving the classification performance. Specifically, this innovative framework integrates KPCA, ISSA, and SVM to fuse sEMG and IMU modalities, with the objective of achieving high-precision recognition of human lower-limb locomotion intent in dynamic environments.

## 2. Method Overview

Human lower-limb locomotion exhibits pronounced rhythmic and periodic characteristics, including fundamental steady-state locomotion modes, such as level walking (LW), stair ascent (SA), stair descent (SD), ramp ascent (RA), and ramp descent (RD), along with dynamic transition models between these primary modes [26]. To enable a more detailed analysis of these locomotion patterns, this study classifies lower-limb locomotion into five distinct steady-state modes and eight transition motion modes based on differences in their kinematic features, as detailed in Table 1.

**Figure 1:**
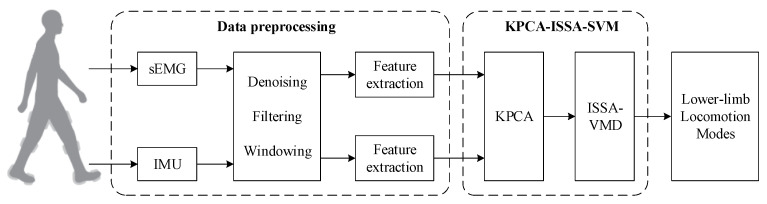
The framework of the proposed lower-limb locomotion intent recognition method.

The framework for lower-limb locomotion intent recognition proposed in this study, as illustrated in Figure 1, consists of four sequential stages: (1) synchronous acquisition of multimodal signals during human locomotion, including sEMG and IMU data; (2) data preprocessing and feature extraction, where raw signals undergo noise reduction, bandpass filtering, and window segmentation, followed by time–frequency domain feature extraction; (3) model training using a hybrid KPCA-ISSA-SVM architecture, which integrates KPCA for nonlinear dimensionality reduction, an ISSA for hyperparameter optimization, and SVM for lower-limb locomotion intent recognition; (4) real-time intent identification by deploying the trained model to classify newly acquired motion signals into predefined locomotion modes (e.g., level walking, stair ascent). The proposed methodology primarily focuses on two technical components. Data preprocessing involves denoising, filtering, and window segmentation applied to raw sensor signals, with subsequent feature extraction to ensure signal integrity and enhance temporal resolution for robust recognition. And the KPCA-ISSA-SVM hybrid algorithm designed to enhance the recognition accuracy through feature space refinement and metaheuristic parameter tuning. Detailed implementations are elaborated in subsequent sections.

## 3. Data Preprocessing

This study proposes a multimodal framework for human lower-limb locomotion intent recognition through the synergistic integration of sEMG and IMU kinematic data. A high-density wireless EMG system (16 channels, 2 kHz sampling rate) was utilized to acquire bilateral lower-limb muscle sEMG, specifically targeting the rectus femoris, tibialis anterior, and soleus muscles during dynamic locomotion tasks. Simultaneously, the IMU sensors were attached on the thigh, shank, and ankle segments to record triaxial acceleration and angular velocity data. The preprocessing pipeline comprised two parallel modules: sEMG feature extraction and kinematic feature extraction.

### 3.1. sEMG Feature Extraction

**Figure 2:**
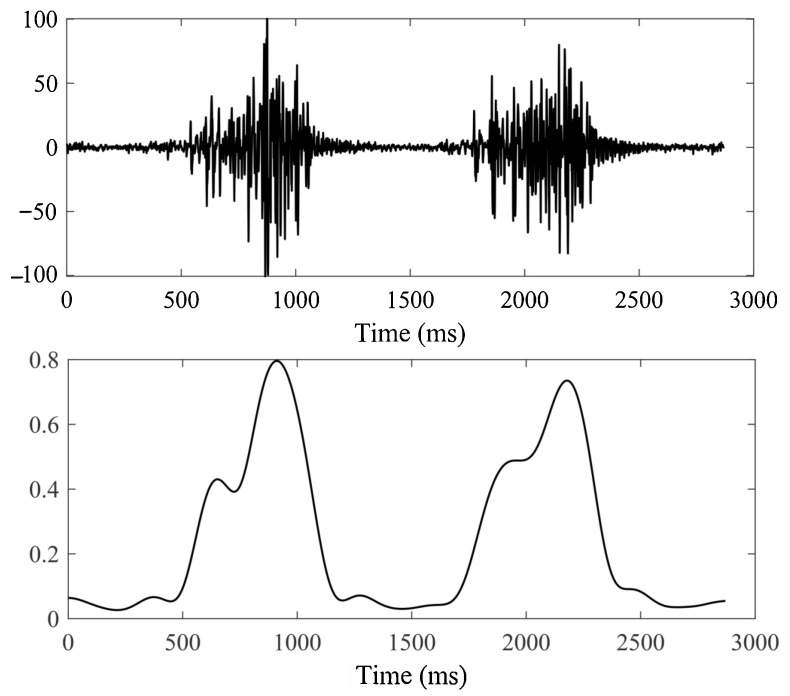
Comparison of sEMG signals before (**above**) and after preprocessing (**below**).

Raw sEMG signals are highly susceptible to exogenous interference, such as ambient electromagnetic noise and motion artefacts, which can significantly compromise the reliability of motion intent recognition [27,28]. To mitigate these disturbances, the acquired sEMG signals were subjected to a sequential preprocessing pipeline: (1) a fourth-order Butterworth bandpass filter with cutoff frequencies of 20 Hz (high-pass) and 250 Hz (low-pass) to reduce baseline drift and high-frequency noise; (2) a 50 Hz notch filter for eliminating powerline interference. The filter stability was verified via a pole-zero analysis, with all poles residing within the unit circle. Furthermore, phase distortion introduced by the Butterworth filter was corrected via forward–backward filtering. The implemented preprocessing pipeline markedly improved the biopotential fidelity of the sEMG signals, as illustrated in Figure 2, which presents a comparison of the sEMG signals before and after preprocessing.

The preprocessed sEMG signals were segmented into consecutive epochs using a 100 ms non-overlapping Hamming window function. Bidomain feature extraction was systematically conducted, with time-domain parameters quantifying amplitude dynamics and frequency-domain descriptors characterising spectral redistribution. The time-domain feature extraction included three amplitude-based metrics that reflect instantaneous muscular activation patterns: the mean absolute value (MAV), which indicates localized contraction intensity; the root mean square (RMS), representing normalized neuromuscular energy expenditure; and the variance (VAR), capturing dynamic force fluctuation magnitudes. Frequency-domain analysis employed spectral metrics, such as the median frequency (MF), which identifies myoelectric spectral compression during fatigue, and the mean power frequency (MPF), quantifying progressive spectral shifts caused by variations in motor unit recruitment rates. Together, these bidomain features form a physiologically interpretable, multidimensional representation of sEMG signal dynamics. The mathematical formulations of the extracted bidomain features are formalized as follows:

(1) $$\mathrm{MAV}=\frac{1}{N}\sum_{i=1}^{N}\left|x_{i}\right|$$

(2) $$\mathrm{RMS}=\sqrt{\frac{1}{N}\sum_{i=1}^{N}{x_{i}}^{2}}$$

(3) $$\mathrm{VAR}=\frac{1}{N-1}\sum_{i=1}^{N}{\left(x_{i}-\bar{x}\right)}^{2}$$

(4) $$\mathrm{MF}=\frac{1}{2}\sum_{j=1}^{M}p_{j}$$

(5) $$\mathrm{MPF}=\frac{\sum_{j=1}^{M}f_{j}p_{j}}{\sum_{j=1}^{M}p_{j}}$$

where $x_{i}$ denotes the discrete signal amplitude at the *k*-*th* sampling point, *N* represents the total number of collected data points, $p_{j}$ indicates the power spectral density at discrete frequency $f_{i}$, and *M* corresponds to the total number of discrete frequency components. The sEMG signals were bilaterally acquired from six lower-limb muscles (three per leg). Five bidomain features were extracted for each sEMG channel; consequently, a 30-dimensional feature vector was constructed by aggregating these statistical measures.

### 3.2. Kinematic Feature Extraction

Three IMU sensors were placed at anatomical landmarks corresponding to the thigh, shank, and ankle to acquire triaxial accelerometry and gyroscopic data during human locomotion. The raw kinematic measurements exhibited significant fluctuations contaminated by high-frequency artifacts arising from soft-tissue oscillations and sensor perturbations. To mitigate these disturbances, a bidirectional fourth-order Butterworth low-pass filter (cutoff frequency: 6 Hz) was implemented for baseline stabilization. Subsequently, the filtered data stream were partitioned into consecutive epochs using a 100 ms non-overlapping Hamming window, which was used to facilitate time-localized feature extraction. Within each temporal window, two statistical descriptors, MAV and VAR, were computed using the Equations (1) and (3). Given that each IMU provides 3D accelerometry and 3D gyroscopic measurements, the feature extraction process generated two statistical descriptors per axis (6 axes × 2 features = 12 features/IMU). Aggregating data from three IMUs yielded a 36-dimensional feature vector through tensor concatenation.

## 4. KPCA-ISSA-SVM

The proposed KPCA-ISSA-SVM framework for lower-limb locomotion intent recognition constructs a comprehensive high-dimensional feature space by integrating synchronized sEMG and IMU data acquisition. KPCA performs nonlinear dimensionality reduction to effectively extract discriminative low-dimensional principal components that retain essential characteristics of lower-limb locomotion modes. Meanwhile, the ISSA algorithm systematically optimizes SVM hyperparameters through iterative convergence criteria, thereby enhancing inter-class separability and ensuring robust generalization across diverse gait cycles.

### 4.1. Kernel Principal Component Analysis

KPCA is a classical nonlinear dimensionality reduction technique based on kernel methods, which effectively reduces data dimensionality and computational complexity while preserving essential features and structural information [29,30]. The core principle of KPCA involves mapping the original data into a high-dimensional feature space ***F*** through a nonlinear transformation, followed by the application of PCA within ***F***. In this study, the feature matrix characterizing lower-limb motion patterns integrates sEMG feature and kinematic feature, formally represented as $X=\left\{x_{1},x_{2},...,x_{n}\right\}$, where *n* denotes the total number of features, each represented by a d-dimensional vector. The nonlinear mapping function ϕ() projects input data into the high-dimensional feature space F, enabling the covariance matrix in feature space F to be formulated as follows:

(6) $$C_{F}=\frac{1}{n}\sum_{i=1}^{n}\phi \left(x_{i}\right)\phi {\left(x_{i}\right)}^{T}$$

Matrix $C_{F}$ is subjected to eigenvalue decomposition:

(7) $$\lambda v=C_{F}v\;$$

where λ denotes the eigenvalue of the covariance matrix $C_{F}$, and v represents its corresponding eigenvector.

Combining Equation (7) into Equation (6) yields the following:

(8) $$C_{F}v=\left(\frac{1}{n}\sum_{i=1}^{n}\phi \left(x_{i}\right)\phi {\left(x_{i}\right)}^{T}\right)v=\frac{1}{n}\sum_{i=1}^{n}\langle \phi (x_{i}),v\rangle \phi \left(x_{i}\right)$$

where $\langle ,\rangle$ denotes the inner product operation. The Equation is equivalent to

(9) $$\lambda \langle \phi (x_{k}),v\rangle =\langle \phi \left(x_{k}\right),C_{F}v\rangle$$

Given the non-vanishing eigenvalue λ≠0, the eigenvector *v* in the high-dimensional feature space admits a linear representation spanned by the mapped samples:

(10) $$v=\sum_{i=1}^{n}a_{i}\phi \left(x_{i}\right)$$

Through the combination of Equations (8)–(10), the following is obtained:

(11) $$\lambda \sum_{j=1}^{n}a_{j}\langle \phi \left(x_{k}\right),\phi \left(x_{j}\right)\rangle =\frac{1}{n}\sum_{j=1}^{n}a_{j}\langle \phi \left(x_{k}\right),\sum_{i=1}^{n}\phi (x_{i})\rangle \langle \phi \left(x_{i}\right),\phi \left(x_{j}\right)\rangle$$

By defining a symmetric kernel matrix K with elements satisfying $K_{\mathrm{ij}}=\phi {\left(x_{i}\right)}^{T}\phi \left(x_{j}\right),$ 1≤i≤n, 1≤j≤n, Equation (11) simplifies to:

(12) nλα=Kα

where $\alpha ={\left[\alpha_{1},\alpha_{2},\ldots ,\alpha_{n}\right]}^{T}$. The eigenvectors of matrix $C_{F}$ are obtained by solving for the linear coefficient α, which also corresponds to an eigenvector of the kernel matrix K. Solving Equations (12) provides the eigenvalues $\lambda_{1}\geq \lambda_{2}\geq \ldots \geq \lambda_{n}$ and associated eigenvectors $\alpha_{1},\alpha_{2},\ldots ,\alpha_{n}$ of K. The contribution rate of each principal component is calculated as follows:

(13) $$C\left(\lambda_{j}\right)=\frac{\lambda_{j}}{\sum_{i=1}^{n}\lambda_{i}}\times 100\%$$

The principal components of the original vectors can be expressed as:

(14) $$t_{k}=\langle v_{k},\phi \left(x\right)\rangle =\sum_{j=1}^{n}a_{j}^{k}\langle \phi \left(x_{j}\right),\phi \left(x\right)\rangle$$

where $t_{k}$ is the *k*-*th* principal component (PC), $v_{k}$ denotes the *k*-*th* eigenvector, $a_{j}^{k}$ represents the coefficient associated with $v_{k}$, and k=1,2…p, *p* is the total number of retained principal components.

KPCA utilizes a kernel function to bypass explicit calculation of nonlinear mappings. The Gaussian radial basis function (RBF) kernel is predominantly selected, defined as follows:

(15) $$K\left(x,x_{i}\right)=\exp\left(-\frac{{∥x-x_{i}∥}^{2}}{2\sigma^{2}}\right)$$

where σ represents the bandwidth parameter of the kernel function, and $∥\cdot ∥$ denotes the Euclidean distance.

### 4.2. Support Vector Machine

This study employs an SVM classifier for lower-limb motion intent recognition. The SVM classification strategy reformulates the problem as a convex quadratic optimization task, aiming to maximize the margin within the feature space [31], and is mathematically expressed as follows:

(16) $$\begin{array}{c} \max\frac{1}{∥\omega ∥};\\ s.t.y_{i}\left(\omega^{T}x_{i}+b\right)\geq 1,i=1,\ldots n. \end{array}$$

where ω denotes the normal vector of the selected hyperplane, $x_{i}$ represents the feature vector, $y_{i}$ corresponds to the classification label, *b* is the threshold parameter, and *n* indicates the total number of training samples. To address linearly inseparable data, SVM employs kernel functions to project samples into a higher-dimensional feature space, where an optimal hyperplane is determined to facilitate linear separation of the mapped samples. The Gaussian radial basis function (RBF) kernel was selected for its capacity to map nonlinear relationships in high-dimensional feature spaces while maintaining computational stability. Its formulation is defined as follows:

(17) $$k\left(x_{1},x_{2}\right)=\exp\left(-g{∥x_{1}-x_{2}∥}^{2}\right),\;g>0$$

where *g* denotes a tunable hyperparameter. To mitigate the impact of outlier-induced classification bias, slack variables $\xi_{i}$ are incorporated into the optimization framework, leading to the reformulated expression of Equation (16):

(18) $$\begin{array}{c} \min\frac{1}{2}{∥\omega ∥}^{2}+C\sum_{i=1}^{n}\xi_{i};\\ s.t.y_{i}\left(\omega^{T}x_{i}+b\right)\geq 1-\xi_{i},\xi_{i}\geq 0,i=1,\ldots ,n \end{array}$$

where *C* denotes the regularization strength, which balances the trade-off between minimizing the empirical error and controlling the model complexity. As shown in Equations (17) and (18), the selection of hyperparameters *C* and *g* fundamentally determines the generalization capability of the SVM classifier. Consequently, this study proposes an improved sparrow search algorithm for the globally optimizing SVM parameters.

### 4.3. Improved Sparrow Search Algorithm

The SSA simulates the positional updating dynamics of sparrow foraging behavior through iterative optimization of individual positions to achieve target objectives [32]. During the search process, sparrows assume three distinct functional roles: explorers, followers and scouts. Compared with conventional optimization algorithms [33,34], SSA offers advantages such as implementation simplicity, rapid convergence speed, and high computational accuracy. However, it exhibits critical limitations, including significant sensitivity to initial parameter settings and a tendency to converge prematurely to local optima, which hinders the attainment of global optimality [35]. To address these deficiencies, this study proposes an enhanced ISSA by incorporating (1) a chaotic opposition-based learning strategy to expand global search capacity; (2) an adaptive dynamic weighting mechanism to balance exploration–exploitation trade-offs; and (3) a hybrid mutation perturbation scheme to circumvent stagnation in local basins. These synergistic modifications collectively mitigate premature convergence while enhancing solution robustness and search diversity.

#### 4.3.1. Population Initialization Based on Chaotic Opposition-Based Learning Strategy

To address the limitations of the standard SSA, where randomly initialized populations often exhibit a heterogeneous distribution and insufficient diversity—thereby negatively impacting the convergence rate and solution accuracy—this study introduces a chaotic opposition-based learning strategy to initialize the sparrow population. This approach improves the stochastic characteristics and diversity of the initial population, ensuring a more uniform spatial distribution across the search space. This enhancement promotes more effective exploration of potential optimal regions and reduces the risk of premature convergence, thereby increasing the likelihood of achieving globally optimal solutions. The initialized population can be formally expressed as follows:

(19) $$X_{\mathrm{ij}}=b_{j}+\left(a_{j}-b_{j}\right)\cdot x_{k}$$

(20) $$x_{k+1}=\;\mathrm{mod}\;\left(x_{k}+c-\frac{d}{2\pi }\sin\left(2\pi x_{k}\right),\;1\right)$$

where $X_{\mathrm{ij}}$ denotes the sparrow position vector; $b_{j}$ and $a_{j}$ represent the lower and upper boundaries of the search space, respectively; $x_{k}$ corresponds to the value of the chaotic mapping sequence; mod(,) is the modulus operator; and c = 0.2 and d = 0.5 are empirically tuned control parameters governing population distribution.

Following chaotic mapping initialization, an opposition-based learning (OBL) strategy is employed to generate the oppositional counterpart population for the current iteration, thereby expanding the exploration coverage of the initialization process. This hybrid mechanism can be mathematically expressed as follows:

(21) $$X_{i,j}^{\mathrm{op}}=a_{j}+b_{j}-X_{\mathrm{ij}}$$

where $X_{i,j}^{\mathrm{op}}$ denotes the position vector of the opposition-based sparrow individual. The opposition population generated by the opposition-based learning strategy and the initial population obtained through chaotic mapping are merged and ranked according to their fitness values. The top 50% of individuals with optimal fitness values are selected to form the refined initial population. This hybrid population initialization strategy ensures a quasi-uniform spatial distribution and enhanced population diversity, thereby improving the initial population quality for subsequent optimization processes.

#### 4.3.2. Explorers’ Position-Updating Strategy

Explorers conduct food source detection within a defined radius, providing directional guidance for the entire population’s foraging behavior. Their positional update equation can be formulated as follows:

(22) $$X_{i,j}^{t+1}=\left\{\begin{array}{cc} X_{i,j}^{t}\exp\left(-\frac{i}{\beta t_{max}}\right) & R<S_{T}\\ X_{i,j}^{t}+QL & R\geq S_{T} \end{array}\right.$$

where $X_{i,j}^{t}$ denotes the position of the *i*-*th* explorer in the *j*-*th* dimension at *t*-*th* iteration, β is a stochastic coefficient uniformly sampled from (0.5,1], $t_{max}$ represents the maximum number of iterations, *Q* is a Gaussian-distributed random perturbation term, L denotes a row matrix with all elements equal to 1 for dimensional consistency, R∈(0.5,1] serves as a real-time risk indicator, and $S_{T}\in \left(0.5,1\right]$ defines the safety threshold. When $R<S_{T}$, explorers engage in routine food-search behavior, iteratively updating their positions toward regions governed by optimized parameter combinations *C* and *g* in SVM. Conversely, if $R\geq S_{T}$, the explorer detects nearby danger, issues a warning to the entire population, and moves to other safe places to forage.

#### 4.3.3. Followers’ Position-Updating Strategy

In the standard SSA, the rapid convergence behavior inherently prevents low-fitness individuals from locating viable food sources, as high-fitness followers aggressively converge toward the perceived optimal region. This excessive exploitation narrows the search domain and reduces population-level precision due to premature stagnation. To address this imbalance between global exploration and local exploitation, a dynamic weighting factor is introduced to adaptively regulate swarm dispersion intensity. The dynamic weighting factor ψ is analytically defined as follows:

(23) $$\psi =\sin\left\{\frac{\pi }{2}\left[1-\frac{\exp\left(\frac{t}{T}\right)-1}{xp\left(1\right)-1}\right]\right\}$$

The update of the followers’ position can be expressed as follows:

(24) $$X_{i,j}^{t+1}=X_{i,j}^{t}+\psi \left|X_{i,j}^{t}-X_{p}^{t+1}\right|$$

where $X_{i,j}^{t+1}$ represents the local optimal position of the explorer in the (t + 1)-th generation.

#### 4.3.4. Scouts’ Position-Updating Strategy

Following position updates of explorers and followers in the SSA, a stochastic vigilance mechanism is activated by randomly selecting 10–20% of the population as scouts, whose position can be expressed as follows:

(25) $$X_{i,j}^{t+1}=\left\{\begin{array}{c} X_{\mathrm{best}}^{t}+\gamma \left|X_{i,j}^{t}-X_{\mathrm{best}}^{t}\right|,\;f_{i}>f_{g}\\ X_{i,j}^{t}+\mu \left(\frac{X_{i,j}^{t}-X_{\mathrm{worst}}^{t}}{\left|f_{i}-f_{w}\right|+\varepsilon }\right),f_{i}=f_{g} \end{array}\right.$$

where γ represents a normally distributed random number, μ denotes a random number within the range [−1, 1], and ε is an infinitesimal constant introduced to prevent division by zero; $f_{i}$ corresponds to the fitness value of the *i*-*th* individual sparrow, $X_{\mathrm{best}}^{t}$ signifies the globally optimal position obtained at the *t*-*th* generation, $X_{\mathrm{worst}}^{t}$ indicates the globally worst position identified at the *t*-*th* generation, while $f_{g}$ and $f_{w}$ characterize the globally optimal fitness value and the worst fitness value achieved during the *t*-*th* generation, respectively.

## 5. Experimentation

### 5.1. Experimental Protocol

In this study, the sEMG and kinematic data were synchronously acquired using an ELONEX EMG100-Ch-Y-RA EMG system (eight channels, sampling frequency: 1000 Hz; Hangzhou Jiaopu Technology Co., Ltd., Hangzhou, China) and six BWT901CL IMUs (Shenzhen Wiite Intelligent Technology Co., Ltd., Shenzhen, China). The sEMG electrodes were placed bilaterally on the rectus femoris, tibialis anterior, and soleus muscles. IMUs were securely mounted using hypoallergenic straps on anatomical landmarks of the thigh (midpoint between the greater trochanter and the lateral femoral condyle), shank (distal third of the tibialis anterior), and ankle segments (lateral malleolus), ensuring minimal motion artifact during dynamic tasks.

Seven healthy participants (mean ± SD: age = 21.2 ± 1.47 years; height = 1.74 ± 0.056 m; body mass = 66 ± 7.9 kg) were recruited for this study. This study was approved by the Institutional Review Board of Pingdingshan University. All participants signed an informed consent form prior to participation, and all collected data were anonymized. Participants completed ten trials of gait analysis across five terrains: (1) level walking, (2) stair ascent, (3) stair descent, (4) ramp ascent (8° inclination), and (5) ramp descent. Each trial incorporated five steady-state locomotion modes and eight transitional modes, as detailed in Table 1, with locomotion performed at self-selected speeds to simulate natural gait kinematics. sEMG signals and IMU data were synchronously recorded. Motion phases were automatically annotated using plantar pressure sensors detecting heel-strike events, with gait cycles normalized to 0–100% duration.

### 5.2. Offline Experimental Results

The proposed KPCA-ISSA-SVM framework was implemented to classify lower-limb motion intents using experimental datasets. To ensure both reproducibility and statistical rigor, a bootstrap-resampling protocol with 100 iterations was applied to the human lower-limb motion dataset. At each iteration, a stratified random sample comprising 80% of the dataset was drawn with replacement to form the training subset, while the remaining 20% was reserved as the held-out test set for performance validation. The classification accuracy was reported as the median value across all bootstrap iterations, with uncertainty quantified through a nonparametric 95% confidence interval (CI) calculated from the 2.5th and 97.5th percentiles of the bootstrap distribution. All model training and testing processes were conducted on a high-performance workstation equipped with an Intel Core i7-10700K CPU (3.80 GHz), 64 GB RAM, and an NVIDIA GeForce RTX 3090 GPU.

**Figure 3:**
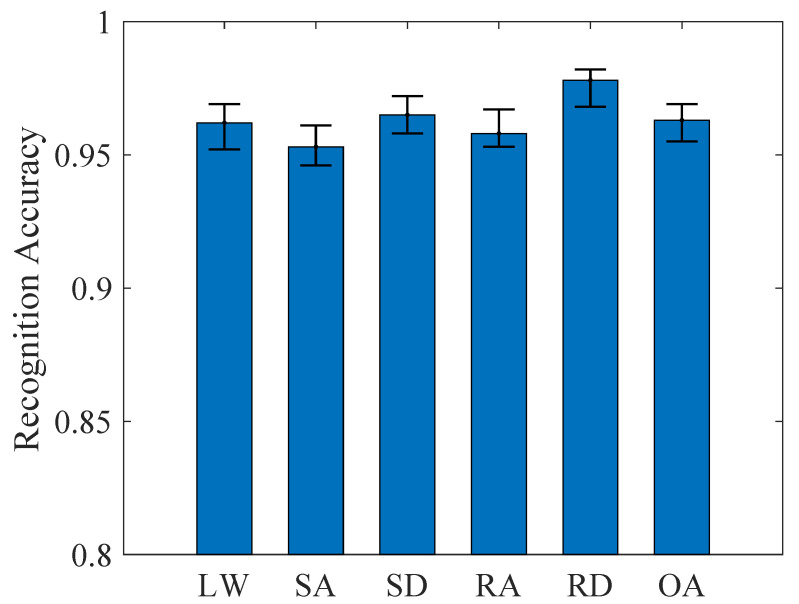
The recognition accuracy of five steady states.

For the five steady-state locomotion modes, the computational cost of this bootstrapping procedure was quantified as 13 ± 4.4 min per iteration on the standard workstation, resulting in a total processing time of 1273 min for 100 iterations. The KPCA-ISSA-SVM framework demonstrated an overall (OA) accuracy of 96.3% (95% CI: 95.5–96.9%), as quantitatively validated in Figure 3. Stair ascent (SA) exhibited the lowest recognition rate of 95.3% (95% CI: 94.6–96.1%), while ramp descent (RD) achieved the highest accuracy of 97.8% (95% CI: 96.8–98.2%). The proposed method shows tighter CIs, indicating higher robustness. A detailed misclassification analysis demonstrated that 2.8% of SA were erroneously classified as RA, and 2.2% of RA were misidentified as SA, likely due to kinematic similarities in lower-limb joint angles and muscle activation patterns between ascent tasks. This observation aligns with prior biomechanical studies highlighting comparable sagittal-plane hip-knee coordination during stair and ramp negotiation [36].

**Figure 4:**
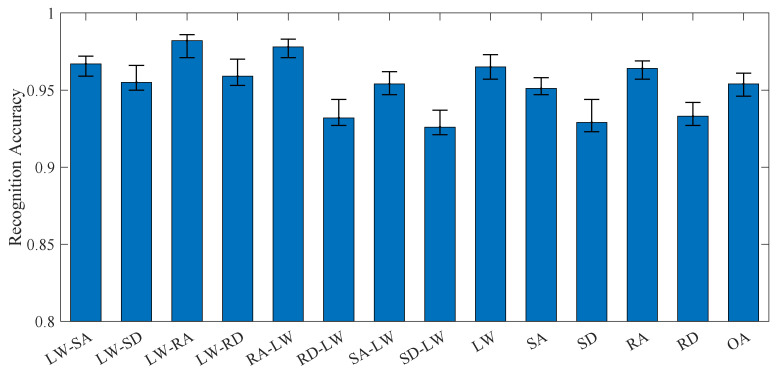
The recognition accuracy under all locomotion modes.

**Figure 5:**
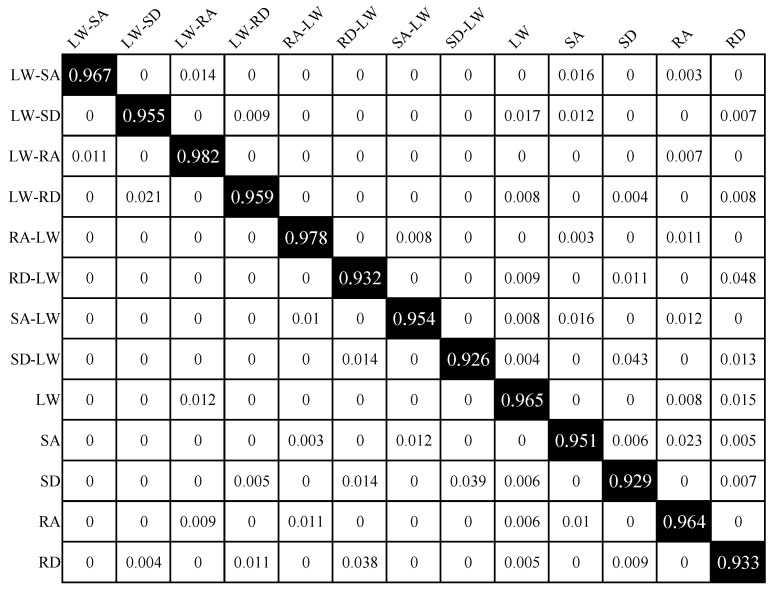
Confusion matrix of all locomotion modes.

For all locomotion modes, each bootstrap iteration completed in 31 ± 5.2 min, resulting in a total processing time of 3146 min for the full bootstrap validation. The KPCA-ISSA-SVM framework achieved OA 95.4% (95% CI: 94.6–96.1%), as quantified in Figure 4. Notably, despite a marginal reduction of 0.97 percentage points compared to its performance under steady-state modes (96.3% OA; 95% CI: 95.5–96.9%), the consistently narrow confidence intervals observed in both scenarios indicate superior algorithmic robustness of the proposed method. The corresponding confusion matrix as shown in Figure 5, which reveals concentrated misclassifications in two specific scenarios: 4.8% of RD-LW samples were misidentified as RD; 4.3% of SD-LW samples were misidentified as SD; 3.9% of SD samples were misidentified as SD-LW; and 3.8% of RD samples were misidentified as RD-LW. All remaining locomotion modes maintained recognition accuracies exceeding 95%. The results demonstrate that RD vs RD-LW and SD vs SD-LW locomotion modes are prone to misclassification. This stems from the complex kinematic signatures and high temporal dynamics inherent in transitional locomotion modes. Specifically, due to the body’s inertial effects, residual kinematic states from the preceding locomotion phase persist into subsequent motion phases, thereby leading to confusion between RD and RD-LW, as well as SD and SD-LW modes.

To simulate real-world noise conditions, we injected additive white Gaussian noise into raw sEMG signals at varying signal-to-noise ratios (SNR: 10 dB, 5 dB, 0 dB). The proposed framework maintained accuracy above 91.2% even at SNR = 5 dB, demonstrating strong noise tolerance. This robustness stems from the bandpass filtering in data preprocessing, and the standardized placement of sEMG sensors is critical to model accuracy. To mitigate this, we recommend using adjustable wearable bands with anatomical markers for consistent sensor positioning. It should be noted that while the proposed KPCA-ISSA-SVM framework achieves high recognition accuracy, its current validation was conducted on a homogeneous cohort of seven healthy young adults, which ensures internal validity for initial algorithmic verification but may limit generalizability to populations with pathological gait patterns (e.g., stroke survivors) or age-related neuromuscular degradation. Future studies should validate this method in larger, clinically diverse cohorts to assess its robustness across varied physiological conditions.

### 5.3. Online Experimental Results

**Figure 6:**
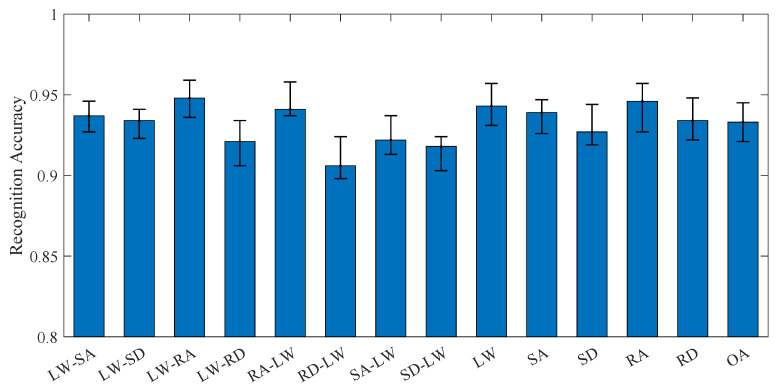
The online experimental recognition accuracy.

Prior to online experimentation, the trained recognition model, optimized through experimental datasets, was deployed onto an NVIDIA Jetson TX2 embedded computing module. During online trials, raw EMG signals and IMU data collected in online operation were transmitted to the TX2 device. A dedicated data-processing pipeline implemented on the TX2 performed real-time preprocessing followed by sliding-window segmentation with a 100 ms non-overlapping Hamming window. The processed feature vectors were subsequently fed into the embedded recognition model for continuous prediction of participants’ motion intent. To ensure experimental consistency, an identical sensor placement protocol (adhesive electrode positions and IMU orientations) was strictly maintained across all seven subjects, as predefined during offline data acquisition. The real-time recognition accuracy is presented in Figure 6, where the bar heights represent the mean recognition accuracy of seven experimental subjects, and the vertical error bars indicate the full data range (minimum to maximum accuracy). The experimental results demonstrate that the proposed online recognition system achieves a robust performance across all motion modes, with the lowest accuracy observed at 90.6% for the SD-LW mode and the highest accuracy reaching 94.6% for the RA mode; the OA accuracy for the 13 locomotion modes is 93.3%. This indicates that the model maintains high reliability in real-time scenarios, despite a slight degradation (2.1%) compared to offline recognition (95.4%). The minor performance decrease can be attributed to online computational constraints, sensor noise, and temporal variations in motion execution.

### 5.4. Comparative Experimental Results

**Figure 7:**
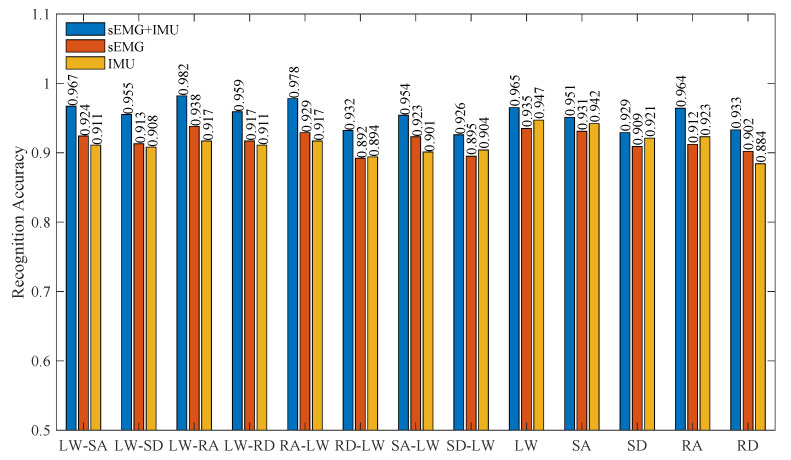
Motion intention recognition accuracy of the proposed method with single-modality data and multimodal fusion.

To validate the superiority of multimodal data fusion based on surface electromyography (sEMG) and inertial measurement unit (IMU) for lower-limb motion intention recognition, this study conducted systematic experiments using the proposed recognition framework under single-modality and fusion configurations. As demonstrated in Figure 7, the recognition accuracy achieved 95.4% in multimodal fusion mode, surpassing the isolated performances of sEMG (91.7%) and IMU (91.4%) alone. This indicates that sensor fusion significantly enhances discriminative power through feature complementarity. For LW, SA, and SD, the IMU-only configuration achieved a competitive accuracy (92.1–94.7%) due to the discriminative kinematic signatures embedded in triaxial accelerometry and angular velocity profiles. This performance discrepancy stems from inherent limitations of individual modalities: sEMG signals, while providing high temporal resolution for neuromuscular activation patterns, exhibit significant inter-subject variability and susceptibility to muscle crosstalk during complex motor tasks, particularly in lower-limb locomotions with similar electrophysiological signatures. Conversely, IMU data effectively capture kinematic trajectories but lack physiological specificity to distinguish motion phases with comparable acceleration profiles. The synergistic fusion mechanism overcomes these constraints through spatiotemporal feature complementarity—sEMG features provide precursor indications of muscle contraction onset, while IMU-derived angular velocities and linear accelerations precisely quantify limb displacement parameters. These results quantitatively confirm that multimodal integration significantly enhances the recognition robustness compared to unimodal approaches, establishing a methodological foundation for clinical applications requiring high-precision motion decoding, such as rehabilitation robotics and neural prosthesis control.

**Figure 8:**
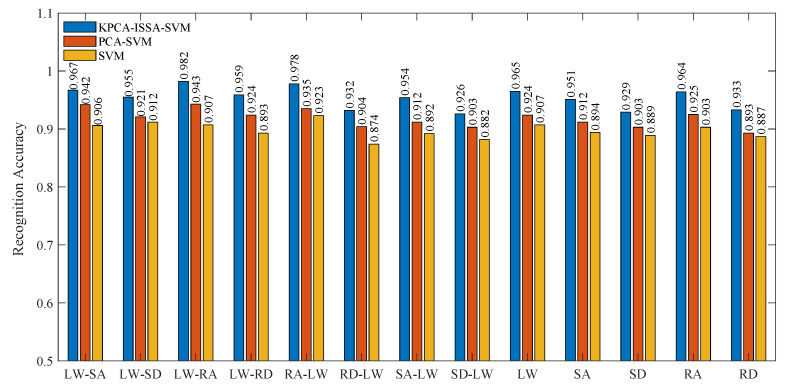
Motion intention recognition accuracy of the different proposed recognition method.

To further validate the effectiveness of the proposed KPCA-ISSA-SVM classification model, comparative experiments were conducted using PCA-SVM and standard SVM algorithms based on experimentally collected multimodal human lower-limb locomotion data for motion intent recognition. As shown in Figure 8, the overall recognition accuracies of PCA-SVM and SVM were 91.85% and 89.76%, respectively, which were 3.50% and 5.59% lower than the proposed KPCA-ISSA-SVM (95.4%). This discrepancy arises because PCA-SVM employs linear principal component analysis (PCA), which struggles to effectively decouple the non-stationarity of sEMG signals from the nonlinear coupling characteristics of IMU dynamic parameters during lower-limb motion. Although PCA-SVM mitigates the “curse of dimensionality” through principal component reduction, its orthogonal decomposition process discards nonlinear, high-order, statistical features critical for classification. This proves that the nonlinear principal components extracted by KPCA are crucial for classification. Additionally, traditional SVM relies on grid search for hyperparameter optimization, which is prone to local optima and computationally inefficient. In contrast, the KPCA in the proposed method maps the original feature space into a high-dimensional separable space via kernel functions, significantly enhancing the discriminability of inter-class boundaries for motion patterns. Meanwhile, the ISSA integrated into the proposed method introduces adaptive inertia weighting and Cauchy mutation strategies, achieving synergistic optimization of penalty factors and kernel parameters during global search. This approach prevents premature convergence and improves the model’s generalization capability.

Although the proposed KPCA-ISSA-SVM framework demonstrates competitive performance in our experiments, emerging deep learning architectures, such as CNNs, LSTM, or transformer models, exhibit promising potential for capturing long-range temporal dependencies in sEMG signals. Nevertheless, their performance heavily depends on large-scale datasets and high computational resources, and these requirements are often unmet in clinical or wearable-device scenarios. A limitation of this study is the baseline comparison scope. While we focused on classical machine learning methods for clinical applicability, future research should evaluate the performance against state-of-the-art deep learning models when larger sEMG datasets become available.

## 6. Conclusions

This study presents a novel KPCA-ISSA-SVM framework for high-accuracy lower-limb motion intent recognition, addressing the critical challenges of nonlinear feature extraction and parameter optimization in multimodal biosignal analysis. By synergistically integrating KPCA for nonlinear dimensionality reduction and an ISSA for SVM hyperparameter tuning, the proposed framework effectively overcomes the limitations of conventional methods in handling complex, high-dimensional sEMG-IMU data. The ISSA incorporates chaotic opposition-based learning, adaptive dynamic weighting, and hybrid mutation strategies, thereby achieving enhanced global search capabilities and mitigating premature convergence risks. Experimental validation on a diverse 13-class locomotion dataset demonstrates a recognition accuracy of 95.35%, surpassing traditional PCA-SVM (91.85%) and standalone SVM (89.76%) benchmarks. Key innovations include the construction of a discriminative low-dimensional feature space through KPCA and the establishment of a robust classification boundary via ISSA-optimized SVM, which collectively enable precise differentiation of steady-state and transitional locomotion modes. Future work should explore real-time deployment in wearable rehabilitation systems and extend validation to clinical populations with mobility impairments. This research establishes a foundational methodology for intention-driven human–machine collaboration, with potential applications in exoskeleton control, smart prosthetics, and neurorehabilitation technologies.

## Figures and Tables

**Table 1:** Thirteen lower-limb locomotion modes and their descriptions.

NO.	Locomotion Mode	Description
1	Level Walking-to-Stair Ascent (LW-SA)	Transition phase from level walking to stair ascent
2	Level Walking-to-Stair Descent (LW-SD)	Transition phase from level walking to stair descent
3	Level Walking-to-Ramp Ascent (LW-RA)	Transition phase from level walking to ramp ascent
4	Level Walking-to-Ramp Descent (LW-RD)	Transition phase from level walking to ramp descent
5	Ramp Ascent-to-Level Walking (RA-LW)	Transition phase from ramp ascent to level walking
6	Ramp Descent-to-Level Walking (RD-LW)	Transition phase from ramp descent to level walking
7	Stair Ascent-to-Level Walking (SA-LW)	Transition phase from stair ascent to level walking
8	Stair Descent-to-Level Walking (SD-LW)	Transition phase from stair descent to level walking
9	Level Walking (LW)	Continuous level walking on flat ground
10	Stair Ascent (SA)	Continuous ascending stairs
11	Stair Descent (SD)	Continuous descending stairs
12	Ramp Ascent (RA)	Continuous ascending slope
13	Ramp Descent (RD)	Continuous descending slope

## Data Availability

Data are unavailable due to privacy or ethical restrictions.

## References

[1] Li L.-L., Cao G.-Z., Liang H.-J., Zhang Y.-P., Cui F. (2023). Human lower limb motion intention recognition for exoskeletons: A review. IEEE Sens. J..

[2] Shi M., Yang C., Zhang D. (2021). A novel human-machine collaboration model of an ankle joint rehabilitation robot driven by eeg signals. Math. Probl. Eng..

[3] Luptáková I.D., Kubovčík M., Pospíchal J. (2022). Wearable sensor-based human activity recognition with transformer model. Sensors.

[4] Semwal V.B., Gupta A., Lalwani P. (2021). An optimized hybrid deep learning model using ensemble learning approach for human walking activities recognition. J. Supercomput..

[5] Ding Z., Yang C., Wang Z., Yin X., Jiang F. (2021). Online adaptive prediction of human motion intention based on semg. Sensors.

[6] Li Z.-Y., Zhao X.-G., Zhang B., Ding Q.-C., Zhang D.-H., Han J.-D. (2021). Review of semg-based motion intent recognition methods in non-ideal conditions. Acta Autom. Sin..

[7] Zhang L., Liu G., Han B., Wang Z., Zhang T. (2019). sEMG based human motion intention recognition. J. Robot..

[8] Semwal V.B., Gaud N., Lalwani P., Bijalwan V., Alok A.K. (2022). Pattern identification of different human joints for different human walking styles using inertial measurement unit (imu) sensor. Artif. Intell..

[9] Gu C., Lin W., He X., Zhang L., Zhang M. (2023). Imu-based motion capture system for rehabilitation applications: A systematic review. Biomim. Intell. Robot..

[10] Li X., Liu J., Huang Y., Wang D., Miao Y. (2022). Human motion pattern recognition and feature extraction: An approach using multi-information fusion. Micromachines.

[11] Bian Q., Castellani M., Shepherd D., Duan J., Ding Z. (2024). Gait intention prediction using a lower-limb musculoskeletal model and long short-term memory neural networks. IEEE Trans. Neural Syst. Rehabil. Eng..

[12] Gao X., Yan L., Wang G., Gerada C. (2021). Hybrid recurrent neural network architecture-based intention recognition for human–robot collaboration. IEEE Trans. Cybern..

[13] Shahini N., Bahrami Z., Sheykhivand S., Marandi S., Danishvar M., Danishvar S., Roosta Y. (2022). Automatically identified eeg signals of movement intention based on cnn network (end-to-end). Electronics.

[14] Yu Z., Lee M. (2015). Human motion based intent recognition using a deep dynamic neural model. Robot. Auton. Syst..

[15] Zhu M., Guan X., Li Z., He L., Wang Z., Cai K. (2023). semg-based lower limb motion prediction using cnn-lstm with improved pca optimization algorithm. J. Bionic Eng..

[16] Zhang T., Sun H., Zou Y. (2022). An electromyography signals-based human-robot collaboration system for human motion intention recognition and realization. Robot. Comput.-Integr. Manuf..

[17] Yin K., Chen J., Xiang K., Pang M., Tang B., Li J., Yang L. (2020). Artificial human balance control by calf muscle activation modelling. IEEE Access.

[18] Embry K.R., Villarreal D.J., Macaluso R.L., Gregg R.D. (2018). Modeling the kinematics of human locomotion over continuously varying speeds and inclines. IEEE Trans. Neural Syst. Rehabil..

[19] Yin K., Li Y., Li X., Zhao H. (2025). Human motion intention recognition via semg and joint kinematics fusion using mpso-svm for intelligent transportation systems. CHAIN.

[20] Wang E., Chen X., Li Y., Fu Z., Huang J. (2024). Lower limb motion intent recognition based on sensor fusion and fuzzy multitask learning. IEEE Trans. Fuzzy Syst..

[21] Liu K., Liu Y., Ji S., Gao C., Zhang S., Fu J. (2023). A novel gait phase recognition method based on dpf-lstm-cnn using wearable inertial sensors. Sensors.

[22] Su B.-Y., Wang J., Liu S.-Q., Sheng M., Jiang J., Xiang K. (2019). A cnn-based method for intent recognition using inertial measurement units and intelligent lower limb prosthesis. IEEE Trans. Neural Syst. Rehabil. Eng..

[23] Tao Y., Huang Y., Zheng J., Chen J., Zhang Z., Guo Y., Li P. Multi-channel semg based human lower limb motion intention recognition method. Proceedings of the 2019 IEEE/ASME International Conference on Advanced Intelligent Mechatronics (AIM).

[24] Tu P., Li J., Wang H. (2024). Lower limb motion recognition with improved svm based on surface electromyography. Sensors.

[25] Yu X., He W., Li Y., Xue C., Li J., Zou J., Yang C. (2019). Bayesian estimation of human impedance and motion intention for human–robot collaboration. IEEE Trans. Cybern..

[26] Su B., Wang J., Liu S., Sheng M., Xiang K. (2020). An improved motion intent recognition method for intelligent lower limb prosthesis driven by inertial motion capture data. Acta Autom. Sin..

[27] Yin K., Xue Y., Yu Y., Xie S. (2021). Variable impedance control for bipedal robot standing balance based on artificial muscle activation model. J. Robot..

[28] Xue Y., Yu Y., Yin K., Du H., Li P., Dai K., Ju Z. (2022). Using adaptive directed acyclic graph for human in-hand motion identification with hybrid surface electromyography and kinect. Symmetry.

[29] Pani A.K. (2022). Nonlinear process monitoring using kernel principal component analysis: A review of the basic and modified techniques with industrial applications. Braz. J. Chem. Eng..

[30] Han Y., Song G., Liu F., Geng Z., Ma B., Xu W. (2022). Fault monitoring using novel adaptive kernel principal component analysis integrating grey relational analysis. Process Saf. Environ. Prot..

[31] Guido R., Ferrisi S., Lofaro D., Conforti D. (2024). An overview on the advancements of support vector machine models in healthcare applications: A review. Information.

[32] Gharehchopogh F.S., Namazi M., Ebrahimi L., Abdollahzadeh B. (2023). Advances in sparrow search algorithm: A comprehensive survey. Arch. Comput. Methods Eng..

[33] Yin K., Tang B., Li M., Zhao H. (2023). A multi-objective optimization approach based on an enhanced particle swarm optimisation algorithm with evolutionary game theory. IEEE Access.

[34] Feng Z., Huang J., Jin S., Wang G., Chen Y. (2022). Artificial intelligence-based multi-objective optimisation for proton exchange membrane fuel cell: A literature review. J. Power Sources.

[35] Ma J., Hao Z., Sun W. (2022). Enhancing sparrow search algorithm via multi-strategies for continuous optimisation problems. Inf. Process. Manag..

[36] Shimotori D., Kato K., Yoshimi T., Kondo I. (2025). Validation of gait kinematics with ramp and stair ascent and descent revealed by markerless motion capture in simulated living space: Test-retest reliability study. JMIR Rehabil. Assist. Technol..

